# Proximal femoral bionic nail is associated with lower postoperative complication risk and better early functional recovery in older patients with intertrochanteric fractures: a retrospective cohort study

**DOI:** 10.1186/s12893-026-03937-6

**Published:** 2026-06-22

**Authors:** Xiaohai Luo, Ning Ma, Yanchuan Yang, Huirong Ma, Ermei Lu, Yuhai Wang

**Affiliations:** Orthopedics Department, People’s Hospital of Ningxia Hui Autonomous Region, 301 Zhengyuan North Street, Yinchuan, Ningxia 750002 China

**Keywords:** Intertrochanteric fracture, Proximal femoral bionic nail, Proximal femoral nail antirotation, Loss of neck-shaft angle, Postoperative complications

## Abstract

**Objective:**

Proximal femoral bionic nail (PFBN) represents a novel biomechanical design for the internal fixation of intertrochanteric fractures in older adults. However, comparative real-world evidence between PFBN and the conventional proximal femoral nail antirotation (PFNA) remains limited, particularly in the presence of baseline differences in patient frailty and bone quality.

**Methods:**

This retrospective study included 301 older patients with intertrochanteric fractures treated between January 2023 and December 2024. According to the fixation method, 201 patients were assigned to the PFNA group and 100 to the PFBN group. Perioperative outcomes, radiographic parameters, composite medical complications, implant-related mechanical complications, and functional recovery were compared between the two groups. The main outcome measures were radiographic stability, composite medical complications, implant-related mechanical complications, and Harris Hip Score (HHS). Although baseline bone mineral density was significantly lower in the PFNA group than in the PFBN group (*P* < 0.001), no statistically significant difference was observed in tip-apex distance (TAD), suggesting broadly similar implant positioning. Multivariable logistic regression was used to adjust for potential confounding factors and to assess the association between implant type and composite medical complications.

**Results:**

The PFBN group had a statistically longer but clinically small difference in operative time compared with the PFNA group (46.97 ± 11.90 min vs. 44.17 ± 9.42 min, *P* = 0.041), whereas intraoperative blood loss and length of hospital stay were similar between the two groups. Radiographic follow-up showed that the mean loss of neck-shaft angle was significantly smaller in the PFBN group than in the PFNA group (0.41 ± 0.57° vs. 2.80 ± 2.31°, *P* < 0.001). During follow-up, implant-related mechanical complications occurred in 3 patients (1.5%) in the PFNA group, all of which were fixation failure events, whereas no implant-related mechanical complication was observed in the PFBN group. Although the crude difference in composite medical complications was not statistically significant, PFBN use was associated with a lower adjusted risk after covariate adjustment (adjusted odds ratio, 0.660; 95% confidence interval, 0.490–0.890; *P* = 0.006). In addition, HHS at 3 and 6 months was modestly higher in the PFBN group, whereas no significant between-group difference was found at 12 months.

**Conclusion:**

Compared with PFNA, PFBN was associated with less loss of neck-shaft angle and a lower adjusted risk of composite medical complications, although the crude comparison of composite medical complications was not statistically significant. Early HHS scores were modestly higher in the PFBN group, but functional outcomes were comparable at 12 months. Given the single-center retrospective design and baseline imbalance, these findings should be interpreted cautiously and require confirmation in prospective multicenter studies.

**Trial registration:**

Not applicable. This was a retrospective cohort study and did not prospectively assign participants to interventions.

**Supplementary Information:**

The online version contains supplementary material available at https://doi.org/10.1186/s12893-026-03937-6.

## Introduction

With the continued acceleration of global population aging, intertrochanteric fractures in older adults have become an important public health concern worldwide and impose a substantial socioeconomic burden on healthcare systems [1–3]. These injuries are often referred to as the “last fracture of life” because they are closely associated with high disability rates and short-term mortality [4–7]. In addition, a study by Kortebein et al. published in JAMA suggested that even a short period of bed rest can lead to significant skeletal muscle loss in healthy older adults, accelerate the progression of sarcopenia, and adversely affect subsequent recovery [8]. Therefore, under the influence of the HIP ATTACK multicenter trial and the concept of enhanced recovery after surgery (ERAS), current orthopaedic management increasingly emphasizes that the goal of surgical treatment is not only to achieve fracture reduction, but also to restore biomechanical stability as early as possible to facilitate early mobilization after surgery [9–11]. Previous clinical studies have shown that frail older patients often have difficulty maintaining restricted partial weight-bearing, and the injured limb is usually subjected to near full weight-bearing once mobilization begins [12]. Accordingly, selecting a stable fixation strategy that better accommodates early weight-bearing is of considerable clinical importance for avoiding the cycle of immobility and postoperative complications.

Cephalomedullary nailing systems, particularly proximal femoral nail antirotation (PFNA), have markedly improved the treatment of unstable intertrochanteric fractures and remain among the most widely used fixation methods in current practice [13, 14]. However, in older and frail patients with substantial osteoporosis, the inherent biomechanical limitations of single-axis load-bearing implants remain a concern [15]. Age-related bone loss may further weaken implant purchase in the proximal femur [16]. As suggested by Baumgaertner’s tip-apex distance (TAD) theory [17], Gotfried’s lateral wall concept [18], and Sommers’ biomechanical model of cut-out [19], impaired cancellous bone quality may substantially reduce the threshold for rotational stability. In the severely osteoporotic proximal femur, postoperative fixation failure, including helical blade cut-out and varus collapse, remains a clinically relevant problem.

To address these biomechanical challenges, a novel proximal femoral bionic nail (PFBN) system has recently been developed based on the concepts of trabecular-mimicking “lever-balance-reconstruction”and triangular support [20]. Unlike conventional intramedullary nails, PFBN incorporates an additional transverse lateral support screw that crosses with the main cephalocervical component. Previous finite element analyses and preliminary clinical studies have suggested that this biomimetic configuration may help distribute mechanical stress more effectively and may improve resistance to rotational instability and varus deformation [21–23]. In addition, a 2025 systematic review and meta-analysis indicated that PFBN may offer potential advantages in fracture healing and early weight-bearing [24].

Despite these preliminary findings, comparative real-world evidence between PFBN and PFNA remains limited. More importantly, observational studies evaluating new orthopaedic implants are often vulnerable to confounding by indication [25]. In routine clinical practice, implant selection may be influenced by baseline patient characteristics, bone quality, and overall frailty. Therefore, methodological guidance, including the STROBE statement, emphasizes the need to identify and transparently report potential confounding factors and to minimize their influence through appropriate statistical adjustment [26]. Otherwise, the true clinical performance of a new intervention may be obscured by crude comparisons.

Therefore, this retrospective cohort study aimed to compare the clinical outcomes of PFBN and PFNA in older patients with hip fractures. We hypothesized that, after adjustment for baseline differences, particularly BMD assessed by DXA, PFBN and PFNA would differ in composite medical complications, radiographic stability, and early functional recovery.

## Methods

### Study design and patient selection

This was a single-center retrospective cohort study conducted at our institution in accordance with the Strengthening the Reporting of Observational Studies in Epidemiology (STROBE) statement. The study protocol was approved by the Institutional Review Board of our hospital (approval No. 2026-LL-050). Because of the retrospective study design, the requirement for written informed consent was waived. All patient data were de-identified before analysis.

We consecutively screened older patients who underwent intramedullary fixation for intertrochanteric fractures at the orthopaedic trauma center of our hospital between January 2023 and December 2024. The predefined inclusion criteria were as follows: (1) age ≥ 65 years; (2) acute closed intertrochanteric femoral fractures caused by low-energy trauma and classified as AO Foundation/Orthopaedic Trauma Association (AO/OTA) type 31-A1 to 31-A3; and (3) treatment with either proximal femoral nail antirotation (PFNA) or proximal femoral bionic nail (PFBN).

The exclusion criteria were as follows: (1) pathological fractures secondary to primary or metastatic bone tumors; (2) severe multiple trauma or open fractures; (3) severe preexisting cognitive impairment or loss of independent ambulation before injury; (4) previous surgery on the ipsilateral hip; and (5) missing key perioperative data or loss to follow-up within 12 months after surgery. Patients with missing key baseline, perioperative, or follow-up outcome data were excluded from the final analysis, and no imputation was performed.

### Surgical procedures and perioperative management

**Fig. 1:**
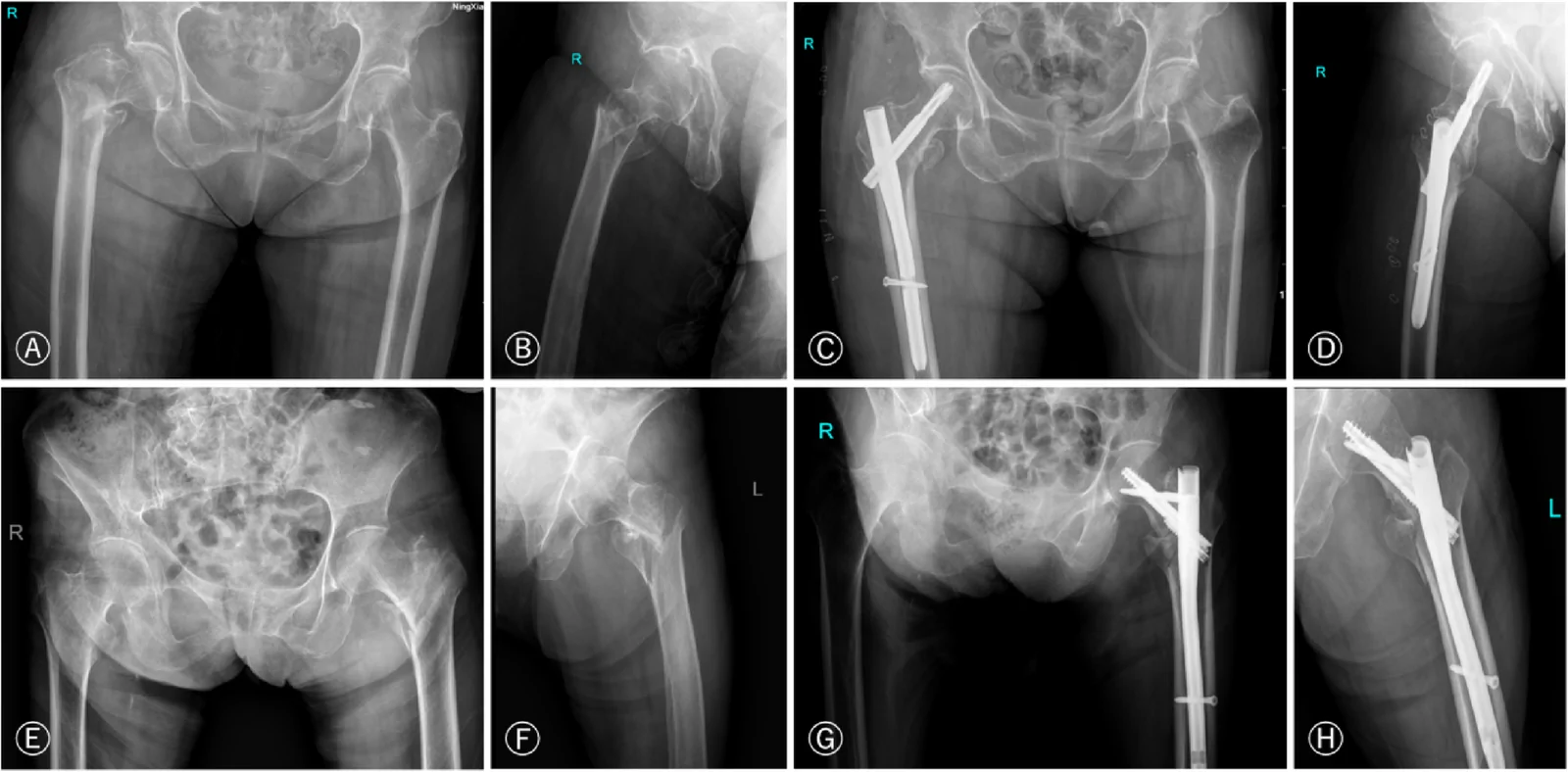
Representative radiographic cases of geriatric intertrochanteric fractures treated with PFNA and PFBN (**A**-**D**) (PFNA group): An 82-year-old female (AO/OTA 31-A2 fracture); (**A**, **B**) are preoperative views, and (**C**, **D**) are the first postoperative radiographs. **E**-**H** (PFBN group): An 85-year-old female (AO/OTA 31-A2 fracture); (**E**, **F**) are preoperative views, and (**G**, **H**) are the first postoperative radiographs demonstrating the bionic triangular support structure

To minimize procedure-related bias, all operations in both groups were performed by the same experienced orthopaedic trauma team. Internal fixation was performed using either a conventional PFNA system (Double Medical Technology Inc., Fujian, China) or a PFBN system (Double Medical Technology Inc., Fujian, China). All procedures were carried out on a traction table under fluoroscopic guidance using standardized techniques. In the PFNA group, the standard operative procedure was followed, with particular attention to accurate placement of the helical blade. In the PFBN group, the implant was inserted according to the manufacturer’s instructions, making use of its specific biomechanical design to provide multiplanar support and improved stability in osteoporotic bone. Representative radiographic images of the two fixation methods are shown in Fig. 1.

Postoperative clinical pathways and rehabilitation protocols were identical in the two groups. Since 2018, our institution has implemented a multidisciplinary fast-track pathway for older patients with hip fractures, through which preoperative evaluation and surgical treatment are routinely completed within 48 h after admission. This standardized enhanced recovery after surgery (ERAS) pathway included uniform prophylactic intravenous antibiotics administered 30 min before surgery and continued for 24 h postoperatively, as well as multimodal analgesia. Prophylaxis for deep vein thrombosis (DVT) was routinely initiated 12 h after surgery using low-molecular-weight heparin and continued until discharge. Under the supervision of trained physiotherapists, all patients were encouraged to begin bedside isometric exercises on postoperative day 1 and then gradually progress to early ambulation and weight-bearing. The timing of weight-bearing was determined by individual pain tolerance and radiographic stability rather than by implant type.

### Data collection and outcome measures

Two independent investigators systematically extracted baseline and clinical data from the electronic medical record system and the picture archiving and communication system (PACS). Baseline covariates included age, sex, body mass index (BMI), fracture classification, preoperative hemoglobin (Hb) level, and preoperative bone mineral density (BMD) measured by dual-energy X-ray absorptiometry (DXA) and expressed as a T-score.

### Primary outcomes

The primary outcomes were radiographic stability, composite medical complications, implant-related mechanical complications, and the trajectory of functional recovery.

Composite medical complications during the 12-month follow-up period included DVT/pulmonary embolism (PE), pulmonary infection, heart failure/myocardial infarction, and pressure ulcer. Implant-related mechanical complications were analyzed separately and included fixation failure, helical blade cut-out, and implant breakage.

Functional recovery was assessed using the Harris Hip Score (HHS) at predefined postoperative time points of 3, 6, and 12 months.

### Secondary outcomes

Secondary outcomes included indicators of surgical quality and perioperative parameters.

Surgical quality was assessed using the tip-apex distance (TAD), which reflects the accuracy of cephalocervical implant placement and was measured on immediate postoperative anteroposterior and lateral radiographs.

Perioperative parameters included operative time (from skin incision to wound closure), estimated intraoperative blood loss, perioperative transfusion requirement, length of hospital stay, and 30-day readmission.

### Statistical analysis

SPSS version 26.0 was used for descriptive statistics and group comparisons. R software version 4.2.2 was used for regression analyses, IPTW sensitivity analysis, and forest plots, with the tableone, WeightIt, cobalt, survey, ggplot2, and forestplot packages. Normality of continuous variables was assessed using the Shapiro-Wilk test. Normally distributed variables are presented as mean ± standard deviation (SD) and were compared using the independent-samples t test. Non-normally distributed variables were compared using the Mann-Whitney U test. Categorical variables are presented as frequencies and percentages and were analyzed using the Pearson chi-square test or Fisher’s exact test, as appropriate. Standardized mean differences (SMDs) were calculated to quantify the degree of baseline imbalance between the two groups, and an SMD > 0.10 was considered to indicate meaningful imbalance. Effect sizes for continuous outcomes were expressed as Cohen’s d.

Given the non-randomized observational design and the anticipated confounding effect of indication bias, particularly the marked baseline imbalance in BMD and sex, we prespecified a multivariable regression strategy. To evaluate the independent association between implant type and composite medical complications, a multivariable logistic regression model was constructed. Variables with a *P* value < 0.10 in the univariable analysis, together with clinically important confounders including BMD, age, sex, BMI, and fracture classification, were forced into the model. Adjusted odds ratios (aORs) and 95% confidence intervals (CIs) were then calculated. The final adjustment set did not include preoperative albumin because it was not part of the prespecified covariate set; therefore, albumin was not reported in the baseline characteristics table.

A multivariable linear regression model was further used to identify factors independently associated with the 6-month HHS. In addition, a prespecified exploratory subgroup analysis of composite medical complications was performed according to age (< 80 years vs. ≥ 80 years), sex, and fracture classification, and the results were presented as forest plots. All statistical tests were two-sided, and a *P* value < 0.05 was considered statistically significant.

To further address baseline imbalance and potential confounding by indication, an inverse probability of treatment weighting (IPTW) sensitivity analysis was performed using propensity scores estimated from age, sex, BMI, BMD, AO/OTA fracture classification, and preoperative Hb. Stabilized weights were applied and trimmed at the 1st and 99th percentiles. Covariate balance before and after IPTW was assessed using SMDs, with an SMD < 0.10 indicating acceptable balance.

## Results

### Patient selection and baseline characteristics

**Fig. 2:**
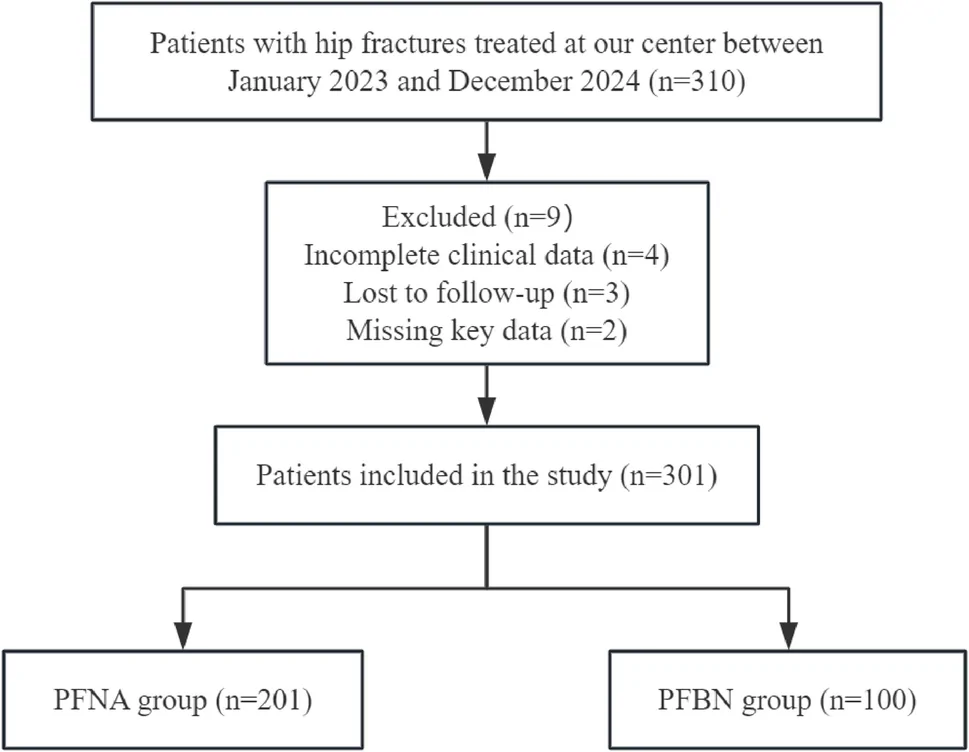
Study flow diagram of patient selection

Between January 2023 and December 2024, a total of 310 patients with hip fractures were initially screened at our center. After exclusion of 9 patients, including 4 with incomplete clinical records, 3 lost to follow-up within 12 months, and 2 with missing key data, 301 patients were included in the final analysis (Fig. 2). All included patients had complete 12-month follow-up data for the predefined clinical and radiographic outcomes. Of these, 201 patients underwent fixation with PFNA and 100 received PFBN.

Baseline demographic and clinical characteristics are summarized in Table 1. No significant between-group differences were observed in mean age (*P* = 0.418), BMI (*P* = 0.067), preoperative Hb level (*P* = 0.354), or fracture classification (*P* = 0.571). However, some baseline imbalance remained between the two groups. The proportion of women was higher in the PFNA group than in the PFBN group (76.1% vs. 62.0%, *P* = 0.016; SMD = 0.313), and preoperative BMD was lower in the PFNA group (-3.537 ± 0.699 vs. -3.162 ± 0.828, *P* < 0.001). The SMD for BMD was 0.504, indicating a substantial imbalance in baseline bone quality; therefore, multivariable adjustment was included in subsequent analyses.

**Table 1:** Baseline characteristics of patients in the PFNA and PFBN groups

Variable	PFNA (*n* = 201)	PFBN (*n* = 100)	*P*-value	SMD
Age (years)	79.741 ± 7.204	80.430 ± 6.395	0.418	0.099
Sex(female)	153/201 (76.1%)	62/100 (62.0%)	0.016	0.313
BMI (kg/m²)	22.364 ± 3.060	23.029 ± 2.756	0.067	0.225
BMD	-3.537 ± 0.699	-3.162 ± 0.828	< 0.001	0.504
Fracture type: A1	52/201 (25.9%)	28/100 (28.0%)	0.571	< 0.001
Fracture type: A2	147/201 (73.1%)	72/100 (72.0%)	0.571	< 0.001
Fracture type: A3	2/201 (1.0%)	0/100 (0.0%)	0.571	0.001
Preoperative Hb (g/L)	104.642 ± 13.569	106.210 ± 14.251	0.354	0.114

In the IPTW sensitivity analysis, baseline covariate balance between the PFNA and PFBN groups was substantially improved after weighting. Before IPTW, meaningful imbalance was observed for BMD, sex, BMI, and preoperative Hb. After IPTW, all included covariates achieved acceptable balance, with SMDs below 0.10. Specifically, the SMDs for BMD and sex decreased from 0.504 to 0.313 before weighting to 0.037 and 0.029 after weighting, respectively. The detailed covariate balance before and after IPTW is shown in Supplementary Table 1.

### Comparison of perioperative outcomes

**Fig. 3:**
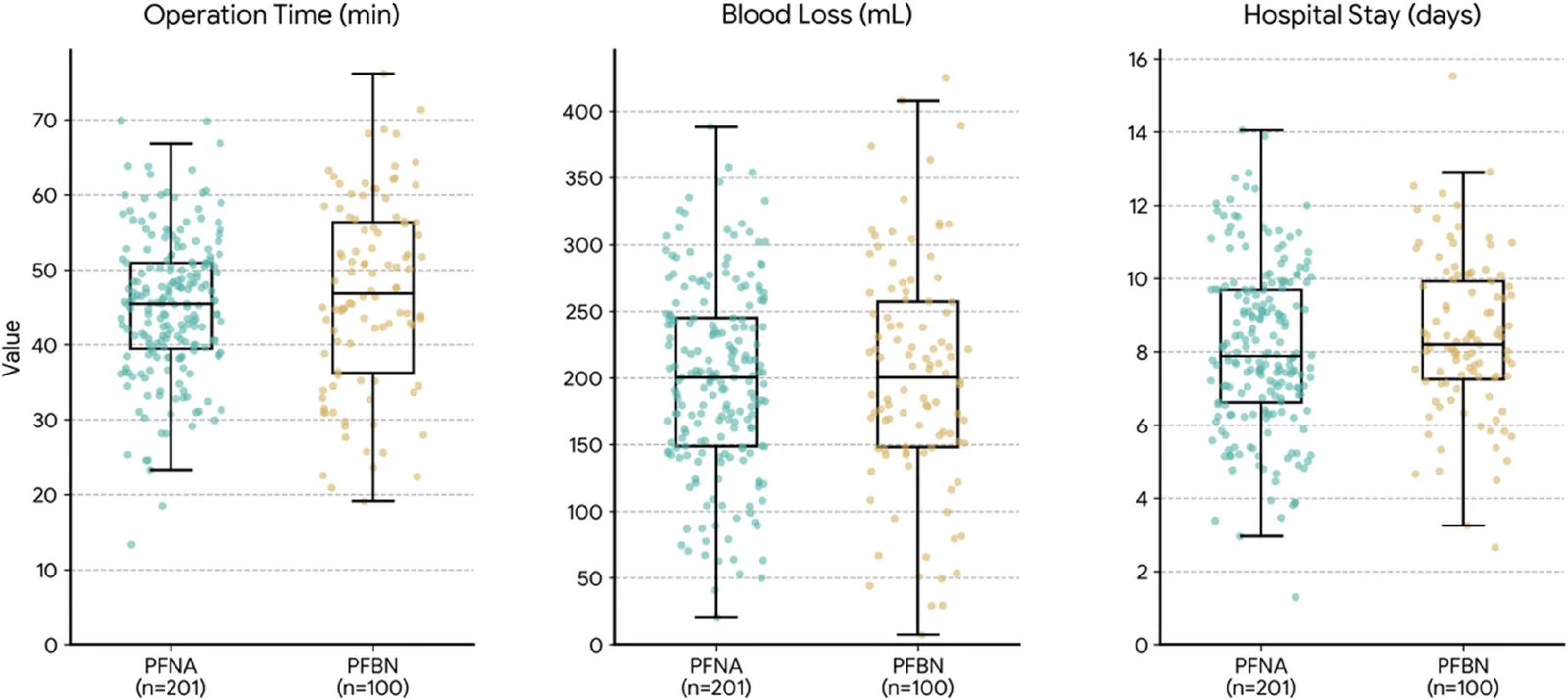
Boxplot comparison of perioperative outcome distributions between the PFNA and PFBN groups

Perioperative outcomes are presented in Table 2 and Fig. 3. The mean operative time was slightly longer in the PFBN group than in the PFNA group (46.97 ± 11.90 min vs. 44.17 ± 9.42 min, *P* = 0.041). However, the absolute between-group difference was small, and the corresponding effect size was also small (Cohen’s d= -0.251), suggesting limited clinical relevance. The remaining perioperative and early recovery-related indicators were generally comparable between the two groups. No significant between-group differences were observed in intraoperative blood loss (194.28 ± 71.80 mL vs. 202.00 ± 75.18 mL, *P* = 0.388), perioperative transfusion rate (60.2% vs. 54.0%, *P* = 0.367), length of hospital stay (7.70 ± 2.25 vs. 7.91 ± 2.11 days, *P* = 0.439), or 30-day readmission rate (3.5% vs. 4.0%, *P* = 1.000).

**Table 2:** Comparison of perioperative outcomes between the PFNA and PFBN groups

Outcome	PFNA (*n* = 201)	PFBN (*n* = 100)	Effect Size	*P*-value
Operation time (min)	44.170 ± 9.423	46.965 ± 11.900	d=-0.251	0.041
Blood loss (mL)	194.279 ± 71.796	202.000 ± 75.183	d=-0.106	0.388
Blood transfusion	121/201 (60.2%)	54/100 (54.0%)	RR = 1.115	0.367
TAD (mm)	17.91 ± 5.30	16.72 ± 5.01	d = 0.342	0.064
Hospital stay (days)	7.701 ± 2.247	7.910 ± 2.113	d=-0.095	0.439
30-day readmission	7/201 (3.5%)	4/100 (4.0%)	RR = 0.871	1.000

### Radiographic assessment, mechanical stability, and fracture healing

Radiographic and mechanical outcomes are summarized in Table 3. With regard to initial surgical quality, the mean postoperative TAD was 17.91 ± 5.30 mm in the PFNA group and 16.72 ± 5.01 mm in the PFBN group, with no statistically significant between-group difference (*P =* 0.064), although definitive equivalence in implant positioning cannot be concluded.

**Table 3:** Radiographic assessment, stability, and implant-related mechanical complications

Variables	PFNA Group (*n* = 201)	PFBN Group (*n* = 100)	*P*-value
Surgical quality			
TAD (mm)	17.91 ± 5.30	16.72 ± 5.01	0.064
Radiological stability			
Loss of neck-shaft angle (°)	2.80 ± 2.31	0.41 ± 0.57	< 0.001
Fracture healing time (months)	4.45 ± 0.82	4.38 ± 0.75	0.468
Fixation failure, n (%)			
Overall fixation failure	3 (1.5%)	0 (0.0%)	0.553
- Helical blade cut-out	2 (1.0%)	0 (0.0%)	-
- Implant breakage	1 (0.5%)	0 (0.0%)	-

**Fig. 4:**
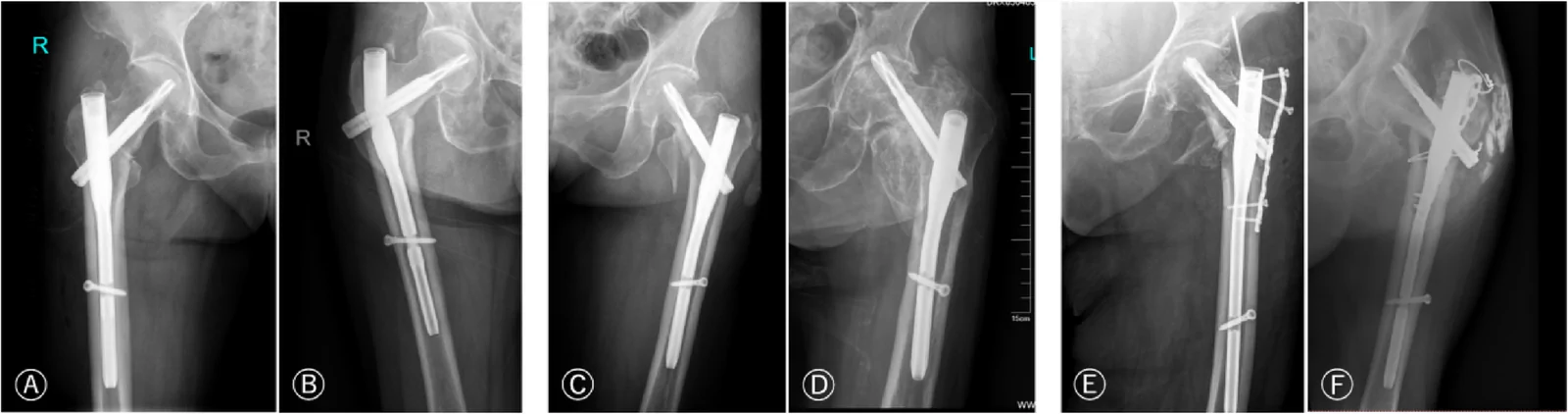
Representative radiographic images of fixation failure in the PFNA group during follow-up (**A**, **B**) An 88-year-old female patient showing helical blade cut-out at 5 months postoperatively; (**C**, **D**) A female patient showing helical blade cut-out at 6 months postoperatively; (**E**, **F**) A 78-year-old female patient showing implant breakage at 4 months postoperatively

Longitudinal radiographic follow-up demonstrated differences in stability between the two groups. The mean loss of neck-shaft angle was smaller in the PFBN group than in the PFNA group (0.41 ± 0.57° vs. 2.80 ± 2.31°, *P* < 0.001). Regarding implant-related mechanical complications, fixation failure occurred in 3 patients (1.5%) in the PFNA group during follow-up, including 2 cases of helical blade cut-out and 1 case of implant breakage (Fig. 4), whereas no fixation failure was observed in the PFBN group. Given the very small number of events, this comparison had limited precision and should be interpreted descriptively. In addition, all patients achieved clinical fracture healing before the final follow-up. The mean fracture healing time was 4.45 ± 0.82 months in the PFNA group and 4.38 ± 0.75 months in the PFBN group, with no significant between-group difference (*P* = 0.468).

### Composite medical complications and multivariable analysis

In the unadjusted analysis (Table 4), the composite medical complication rate was numerically lower in the PFBN group than in the PFNA group (25.0% [25/100] vs. 33.8% [68/201]); however, this difference did not reach statistical significance (RR = 1.353, 95% CI 0.916–2.000, *P* = 0.153). Similarly, no significant between-group differences were observed in specific complications, including DVT/PE and pulmonary infection (all *P* > 0.05).

**Table 4:** Comparison of composite medical complications between the PFNA and PFBN groups

Complication Type	PFNA (*n* = 201)	PFBN (*n* = 100)	RR (95% CI)	*P*-value
Composite medical complications	68/201 (33.8%)	25/100 (25.0%)	1.353(0.916-2.000)	0.153
DVT/PE	52/201 (25.9%)	22/100 (22.0%)	1.176(0.760–1.820)	0.554
Pulmonary infection	15/201 (7.5%)	4/100 (4.0%)	1.866(0.636–5.475)	0.318
Heart failure/MI	4/201 (2.0%)	0/100 (0.0%)	-	0.306
Pressure ulcer	0/201 (0.0%)	1/100 (1.0%)	-	0.332

**Fig. 5:**
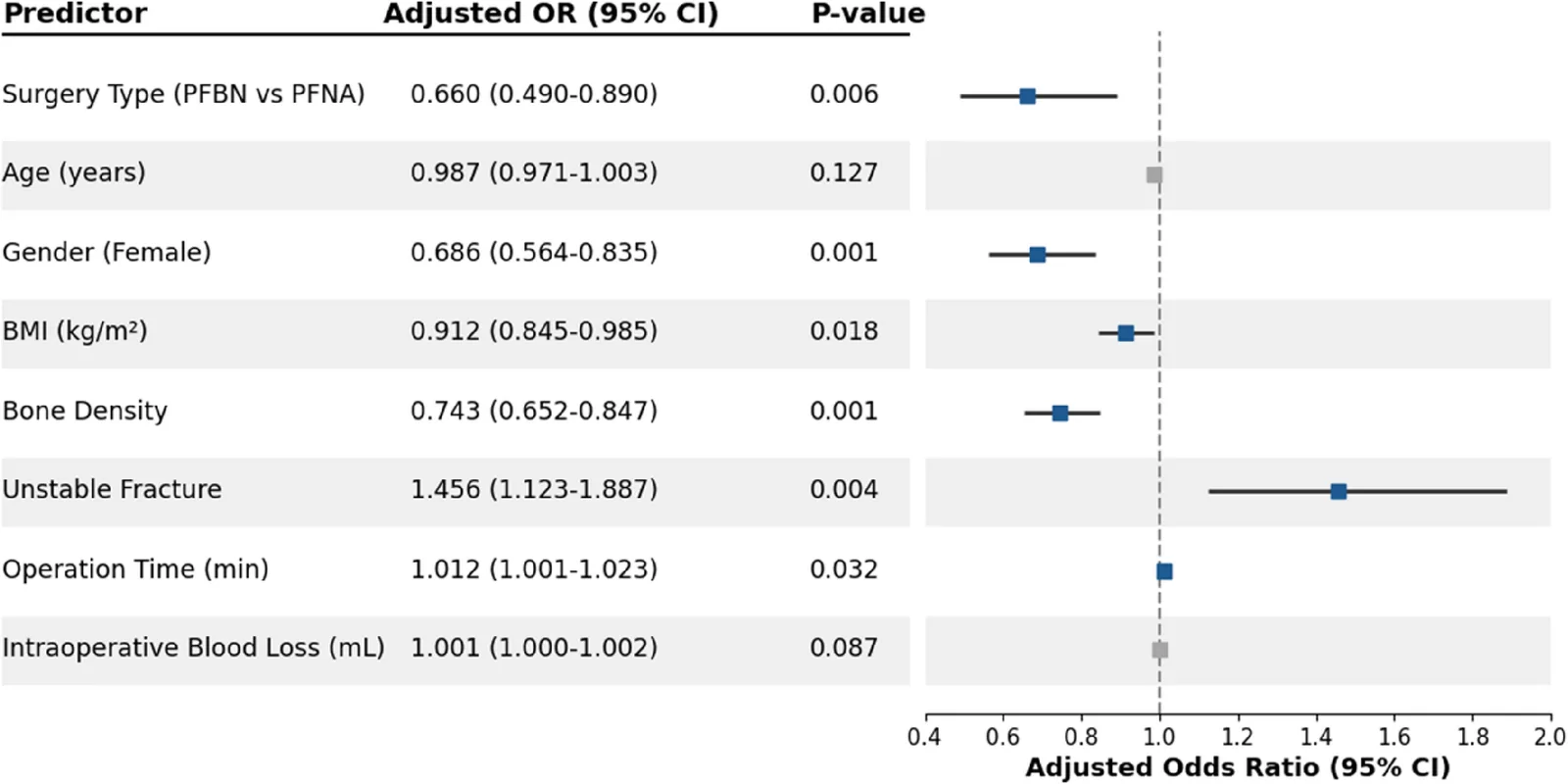
Forest plot of multivariable logistic regression analysis for independent predictors of composite medical complications

Because of the baseline imbalance, particularly in BMD, a multivariable logistic regression model was further constructed to adjust for potential confounding factors. The results are shown in the forest plot in Fig. 5. After adjustment for baseline covariates, PFBN use was associated with a lower adjusted risk of composite medical complications (adjusted OR = 0.660, 95% CI 0.490–0.890, *P* = 0.006). In addition, Fig. 5 shows that female sex, higher BMI, and higher BMD were associated with a lower risk of composite medical complications, whereas unstable fracture pattern and longer operative time were associated with a higher risk.

### Functional recovery over time

**Fig. 6:**
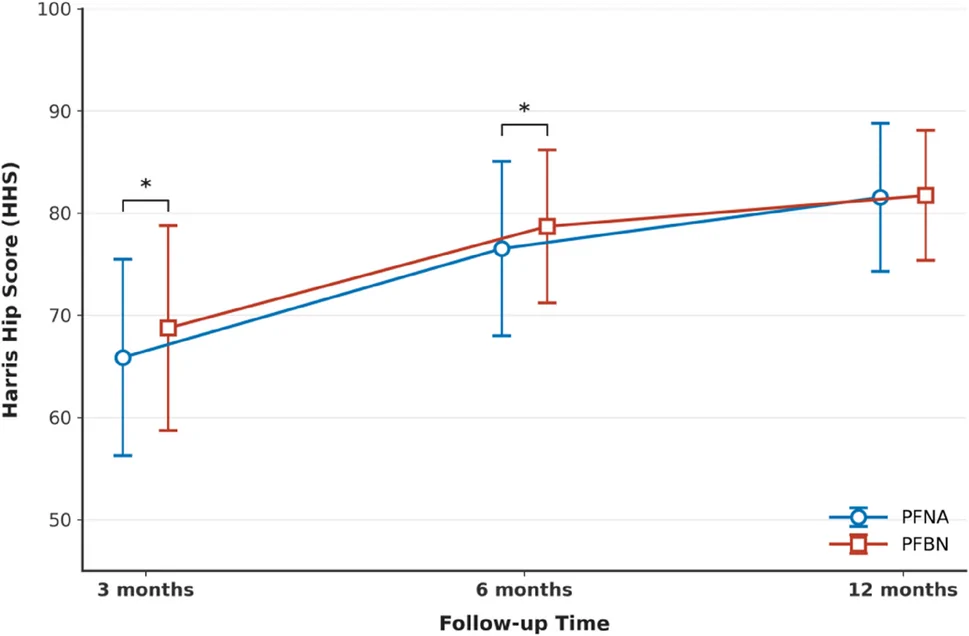
Longitudinal comparison of functional recovery based on Harris Hip Score (HHS) between the PFNA and PFBN groups

Longitudinal postoperative hip function assessed by HHS showed a pattern of earlier between-group differences and later convergence (Fig. 6). During the early recovery period, HHS at 3 and 6 months was higher in the PFBN group than in the PFNA group (both *P* < 0.05), whereas no significant between-group difference was observed at 12 months (Table 5). To further identify factors associated with early functional recovery, a multivariable linear regression analysis was performed using the 6-month HHS as the dependent variable (Table 6). PFBN fixation (β = 2.100, 95% CI 1.301–2.899, *P* = 0.001), as well as higher preoperative Hb and BMD, was associated with a higher 6-month HHS. In contrast, older age and longer operative time were associated with a lower 6-month HHS.

**Table 5:** Comparison of functional recovery during follow-up between the PFNA and PFBN groups

Time Point	PFNA HHS	PFBN HHS	MD	95% CI	*P*-value
3 months	65.857 ± 9.612	68.733 ± 10.021	-2.876	(-5.224, -0.528)	0.017
6 months	76.508 ± 8.542	78.683 ± 7.481	-2.175	(-4.151, -0.199)	0.031
12 months	81.531 ± 7.239	81.715 ± 6.367	-0.184	(-1.861, 1.493)	0.829

**Table 6:** Multivariable linear regression analysis of factors associated with 6-month Harris Hip Score

Predictor	Coefficient	Standard Error	95% CI	*P*-value
Surgery type (PFBN vs. PFNA)	2.100	0.854	(1.301, 2.899)	0.001
Age (years)	-0.156	0.067	(-0.288, -0.024)	0.021
Gender (Female)	1.234	0.723	(-0.187, 2.655)	0.088
Preoperative Hb (g/L)	0.456	0.089	(0.281, 0.631)	0.001
BMD	2.789	0.912	(0.998, 4.580)	0.002
Operation time (min)	-0.078	0.034	(-0.145, -0.011)	0.023
Intraoperative blood loss (mL)	-0.006	0.003	(-0.012, 0.000)	0.056

### Exploratory subgroup analysis of composite medical complications

**Fig. 7:**
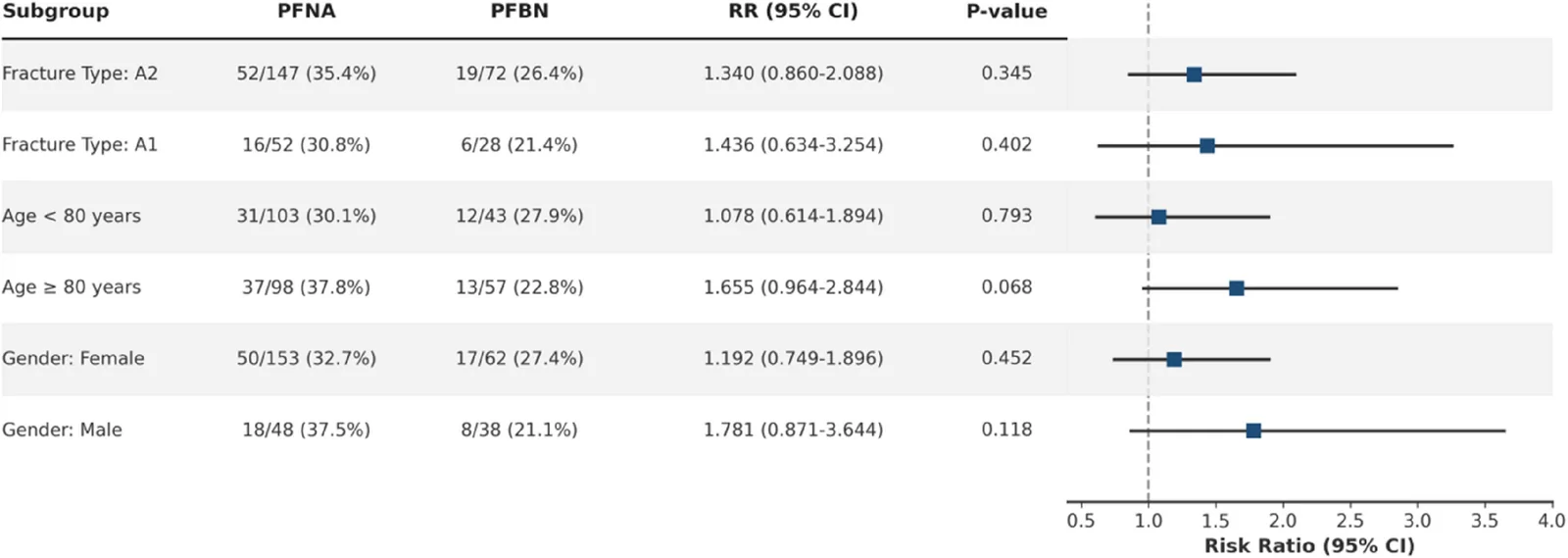
Forest plot of exploratory subgroup analysis for composite medical complications

To further explore potential risk patterns across different patient groups and fracture types, an exploratory subgroup analysis of composite medical complications was performed (Fig. 7). In all subgroups, the complication risk was numerically lower in the PFBN group than in the PFNA group. Although several comparisons did not reach statistical significance because of the reduced sample size in subgroup analyses, the relative risk estimates for PFNA were higher in very old patients (≥ 80 years; RR = 1.655, 95% CI 0.964–2.844, *P* = 0.068) and in male patients (RR = 1.781, 95% CI 0.871–3.644, *P* = 0.118). Because of the limited sample size and wide confidence intervals in the subgroup analyses, these findings should be considered exploratory and hypothesis-generating only.

## Discussion

The main finding of this retrospective cohort study was that PFBN was generally comparable with PFNA in terms of perioperative burden but was associated with less postoperative loss of neck-shaft angle and modestly better early functional recovery. Although the crude composite medical complication rate did not differ significantly between the two groups, PFBN use was associated with a lower adjusted risk of composite medical complications after accounting for baseline imbalance, particularly the lower BMD in the PFNA group. Therefore, this finding should be interpreted as an adjusted association rather than evidence of a causal effect.

### Statistical interpretation of the complication findings

One notable finding in our cohort was the discrepancy between the unadjusted and adjusted analyses of composite medical complications. The lack of a significant crude difference may partly reflect baseline imbalance and confounding by indication, which are common in observational studies [25]. In clinical practice, implant selection may be influenced by patient condition, bone quality, fracture morphology, and surgeon preference. In our study, the PFNA group had lower baseline BMD, and reduced bone structural density may affect cephalomedullary nail stability and increase the risk of fixation-related failure [17, 19, 27].

Current epidemiological and methodological guidance emphasizes the importance of controlling for baseline imbalance in observational research through appropriate statistical modeling [26]. In the present study, the association between PFBN use and a lower adjusted risk of composite medical complications became apparent after adjustment for key confounders, including BMD. This finding highlights the importance of adequately accounting for baseline bone quality when evaluating the real-world performance of new orthopaedic implants.

### Biomechanical considerations, surgical burden, and early mobilization

Although the operative time differed slightly between the two groups, the absolute difference was small and unlikely to be clinically meaningful. Blood loss and transfusion rates were similar, suggesting broadly comparable perioperative burden [28].

From a radiographic perspective, PFBN was associated with less loss of neck-shaft angle during follow-up. Although postoperative TAD was within an acceptable range in both groups and did not differ statistically, definitive equivalence in implant positioning cannot be concluded because of the numerical between-group difference. The greater loss of neck-shaft angle in the PFNA group suggests that maintenance of reduction may differ between the two constructs. PFNA relies on a single-axis load-bearing mechanism and can be mechanically compared to a cantilever-like structure. Under repetitive loading in older patients with severe osteoporosis, its resistance to varus deformation may be limited, which may contribute to progressive varus collapse. In the present study, implant-related mechanical complications were observed in 3 patients in the PFNA group, all of which were fixation failure events, and in none of the PFBN group. However, because the total number of events was very small, this comparison was statistically underpowered and had limited precision. Therefore, it should be interpreted as a descriptive finding rather than evidence of mechanical superiority.

The postoperative functional trajectory in our study showed earlier benefit and later convergence. HHS was higher in the PFBN group at both 3 and 6 months, which may be related to improved early mechanical stability provided by its biomimetic structure. However, the absolute between-group differences in HHS at 3 and 6 months were modest, approximately 2–3 points. Therefore, although statistically significant, these differences may not necessarily exceed the minimal clinically important difference and should be interpreted cautiously. A finite element analysis published in 2025 reported that PFBN forms a multiplanar triangular stabilizing construct through a crossed lateral support screw and may distribute peak von Mises stress more effectively while reducing tangential micromotion at the fracture site compared with conventional single-axis intramedullary fixation [29].

Clinically, one possible explanation is that improved construct stability may facilitate early rehabilitation; however, postoperative loading, rehabilitation adherence, and objective mobility performance were not directly measured in this study. Previous studies have suggested that weight-bearing and postoperative mobility are important for functional recovery and prognosis after hip fracture surgery [30, 31]. Therefore, the relationship between PFBN design, early mobilization, and composite medical complications remains speculative.

### Clinical relevance in higher-risk subgroups

The subgroup analyses were limited by small sample sizes and wide confidence intervals. Therefore, the observed directional trends in male patients and very old patients should be interpreted only as exploratory and hypothesis-generating findings, rather than evidence of subgroup-specific clinical benefit.

### Limitations

This study has several limitations. First, as a single-center retrospective study, residual confounding could not be fully excluded despite multivariable adjustment and IPTW sensitivity analysis. In particular, surgeon preference, fracture reduction quality, frailty status, cognitive function, and rehabilitation adherence were not fully captured in the retrospective database. Second, the PFBN group had a relatively limited sample size, and the number of fixation failure events was very small, which limited the statistical power and precision of subgroup and mechanical safety analyses. Third, although a 1-year follow-up period is sufficient to assess fracture healing and short-term complications, it is not adequate to evaluate longer-term implant survival. Prospective multicenter randomized studies are still needed to further validate the present findings.

## Conclusion

Compared with PFNA, PFBN was associated with less loss of neck-shaft angle and a lower adjusted risk of composite medical complications, although the crude comparison of composite medical complications was not statistically significant. Early HHS scores were modestly higher in the PFBN group, but functional outcomes were comparable at 12 months. Given the single-center retrospective design and baseline imbalance, these findings should be interpreted cautiously and require confirmation in prospective multicenter studies.

## Supplementary Information

Supplementary Material 1.

## Data Availability

The datasets used and/or analyzed during the current study are available from the corresponding author on reasonable request.
