# Supplementary material for: Proximal femoral bionic nail is associated with lower postoperative complication risk and better early functional recovery in older patients with intertrochanteric fractures: a retrospective cohort study
Source: BMC Surg. 2026 Jun 22;26:605. doi: 10.1186/s12893-026-03937-6 (PMC13560243; doi:10.1186/s12893-026-03937-6)
Supplement: Supplementary file 1 [file 12893_2026_3937_MOESM1_ESM.docx]

**Supplementary Table 1** Covariate balance before and after inverse probability of treatment weighting

| Variable | SMD before IPTW | SMD after IPTW |
| --- | --- | --- |
| Age (years) | 0.099 | 0.083 |
| Sex(female) | 0.313 | 0.029 |
| BMI (kg/m²) | 0.225 | 0.030 |
| BMD | 0.504 | 0.037 |
| AO/OTA fracture pattern, A2/A3 vs A1 | 0.048 | 0.065 |
| Preoperative Hb (g/L) | 0.114 | 0.097 |

**Abbreviations:** IPTW, inverse probability of treatment weighting; SMD, standardized mean difference; BMI, body mass index; BMD, bone mineral density; AO/OTA, AO Foundation/Orthopaedic Trauma Association; Hb, hemoglobin. Stabilized weights were trimmed at the 1st and 99th percentiles. An SMD < 0.10 was considered indicative of acceptable balance.
